# Effectiveness of a Web-Based Virtual Simulation to Train Nursing Students in Suicide Risk Assessment: Randomized Controlled Investigation

**DOI:** 10.2196/69347

**Published:** 2025-08-01

**Authors:** Paul Roux, Yujiro Okuya, Cristina Morel, Mariane Soulès, Hugo Bottemanne, Eric Brunet-Gouet, Solène Frileux, Christine Passerieux, Nadia Younes, Jean Claude Martin

**Affiliations:** 1 DisAP - MOODS Team, INSERM UMR1018, CESP, Université de Versailles Saint-Quentin-En-Yvelines - Université Paris-Saclay, Le Chesnay, France; 2 Service Universitaire de Psychiatrie d'Adultes et d’Addictologie, Centre Hospitalier de Versailles, 177 rue de Versailles, Le Chesnay, 78150, France, 33 0139639380; 3 Laboratoire Interdisciplinaire des Sciences du Numérique, CNRS, Université Paris-Saclay, Orsay, France; 4 Institut de Formation en Soins Infirmiers de Versailles, Centre Hospitalier de Versailles, Versailles, France; 5 Institut de Formation en Soins Infirmiers de Rambouillet, Centre Hospitalier de Rambouillet, France; 6 Service Hospitalo-Universitaire de Psychiatrie, Hôpital Bicêtre, Assistance Publique - Hôpitaux de Paris, Le Kremlin-Bicêtre, France; 7 MOODS Team, INSERM UMR1018, CESP, Faculté de Médecine Paris-Saclay, Université de Versailles Saint-Quentin-En-Yvelines, Université Paris-Saclay, Villejuif, France; 8 Equipe DevPsy, INSERM UMR1018, Centre de recherche en Épidémiologie et Santé des Populations, Université Paris-Saclay & Université Versailles Saint-Quentin-En-Yvelines, Villejuif, France

**Keywords:** nurse, simulation, virtual patient, suicide, depression, education, confidence, emotion, prosody, facial expression

## Abstract

**Background:**

Suicide is a leading cause of preventable death worldwide. Nurses play a critical role in suicide prevention; yet, they face significant obstacles. Improving the evaluation and management of patients at risk of suicide requires innovative training techniques that safely and effectively enhance nursing students’ skills, knowledge, and confidence. Virtual simulation (VS) based training can be particularly effective because it allows interaction with patients without the risk of causing harm.

**Objective:**

The purpose of this study was to evaluate the pedagogical effectiveness of a novel VS tool featuring a fully automated and emotionally reactive virtual patient by assessing its ability to assist nursing students in learning suicide risk assessment. VS also included an online group debriefing, co-run by a nurse and a medical teacher.

**Methods:**

A randomized controlled investigation was conducted with 68 first-year nursing students recruited from nursing schools offline and online. They were divided into a control group receiving teaching as usual (TAU) and an intervention group receiving TAU plus VS. The intervention was purely web-based and unblinded. Outcomes were self-assessed through questionnaires using Kirkpatrick Training Evaluation Model, which focuses on knowledge, skills, confidence, empathy, and satisfaction among students.

**Results:**

The VS group exhibited significantly higher confidence (3 points of increase after TAU vs 10.6 points of increase after VS, B=7.2; SE 2.5; *t*_111.5_=2.8; *P*=.006) and a marginally enhanced ability to respond appropriately to suicidal thoughts (1.6 points of improvement after TAU vs 6.4 points of improvement after VS, B=−4.5; SE 2.5; *t*_119.5_=−1.8; *P*=.08) compared with the control group. However, there were no significant differences in knowledge acquisition or the general level of empathy. Satisfaction with VS was high, particularly regarding the authenticity of the virtual patient. Authenticity was perceived as greater when emotional prosody was included with facial emotions.

**Conclusions:**

The use of VS demonstrated promising results in enhancing nursing students’ confidence in detecting suicide risk and their skills in counseling individuals experiencing a suicide crisis, suggesting its incorporation into routine teaching methods. Further research is needed to explore its long-term benefits for students and its impact on patient outcomes.

## Introduction

Suicide is among the leading causes of death worldwide, with 703,000 deaths by suicide in 2019, which was more than deaths due to malaria, HIV, breast cancer, war, and homicide [1]. One important axis of suicide prevention is the education of health care professionals in recognizing depression and managing suicidal crises. However, this aspect requires further investigation to assess its possible benefits due to the small number of randomized controlled investigations [2].

Nurses are ideally positioned to intervene and implement preventative measures toward suicide. However, several obstacles in suicide risk evaluation and care by nurses have been identified. First, negative attitudes and a stigma toward patients experiencing suicide crises are high among nurses, although less frequent than among doctors [3]. Second, nurses do not usually use guidelines or suicide assessment instruments when they evaluate suicidality [4,5], which may result from deficits in skill and knowledge [6]. Gaps in the training of nurses have been reported by psychiatric nurses associations in the management of individuals with suicidal ideation [7], and nursing students reported a lack of education and knowledge in suicide prevention [8]. Some qualified nurses also experience not having sufficient knowledge or skills to feel safe in suicide detection [9] and, sometimes, avoid asking questions about suicidality for fear of what to do with the answer [10]. One of the major obstacles in suicide prevention may, thus, result from a lack of confidence. Health sciences students are afraid of talking about suicide with patients [11] in particular, asking direct questions about suicidal thoughts and intentions, as they believe such questions could embarrass or distress patients. To decrease such clinical anxiety, health students are usually taught that, according to the literature, talking about suicide within a screening interview does not increase distress or suicide ideation by patients [12,13] and may even improve their sense of well-being [14]. However, students often doubt that a patient with suicidal intent can feel relief when speaking about suicide until they experience it during a face-to-face interaction. It, therefore, seems necessary for students to experience the relief felt by patients with suicidal ideation when they are interviewed about their suicidal thoughts: this would make it easier for them to put into practice their competence in suicide prevention with real patients. It would also be safer if these first experiences did not occur with real patients.

Usual teaching methods have shown pedagogical effectiveness for suicide prevention in nursing studies. Short lectures and discussions of health care staff experiences and videotaped testimonials by experts on the lived experience of suicidal crises have been shown to be associated with an increase in self-perceived competence [15]. A 3.5-hour suicide prevention training course with video clips, practicing skills, and group discussion was associated with a significant increase in general perceived self-efficacy compared to preintervention [16]. However, some data suggest that simulation may be more efficient than usual teaching methods to improve suicide risk detection and care [17]. The most prominent simulation method in psychiatry involves standardized patients [18], played by professional actors, teachers, or carers. However, it is often difficult and costly to find, train, pay, and debrief standardized patients, thus limiting the spread of this teaching method. By contrast, simulation with fully autonomous virtual patients allows perfect standardization of the environment, a potentially infinite number of learners for one simulation, and remote learning. Randomized controlled studies of the pedagogical efficiency of virtual simulation (VS) targeting suicide risk are scarce. Only one study involving second-year medical students reported that students who benefited from VS inquired more frequently than students who benefited from a video-based intervention in several suicide risk areas during an interaction with a simulated patient [19]. However, no differences were found in communication skills, such as the learner’s professional appearance, behavior, and empathy. The virtual patient consisted of an embodied conversational agent portrayed by a static image of her face, without any emotional reaction to the questions asked by the learner. This may have lacked realism, as interpreting subtle nonverbal cues a patient displays is a critical skill for health students. During a psychiatric interview, facial expressions can provide an understanding of the patient’s affective processes [20]. Thus, autonomous and emotionally reactive virtual patients are needed to simulate nursing interviews in suicidology teaching properly.

The present study aimed to evaluate the pedagogical effectiveness of a complementary simulation teaching using an autonomous and emotionally reactive virtual patient (Simulation with a Virtual Patient in Psychiatry [SIVIPSY]) compared with traditional teaching alone. We made the hypothesis that this teaching tool would help nursing students to learn how to detect and evaluate a suicidal crisis and behave appropriately. The study also aimed to establish the degree of perceived realism of VS and the impact of the virtual patient’s emotional reactions on the realism of SIVIPSY and learner satisfaction.

## Methods

### Design of the Virtual Patient

SIVIPSY consisted of a VS of a nurse interview with a patient experiencing severe depression with an imminent suicide risk [21]. It was designed by nursing teachers, academic psychiatrists, and computer science researchers specializing in human-computer interactions. In SIVIPSY, learners must dialogue with an emotionally reactive avatar according to an underlying decision tree architecture: they have to select the best question to ask, which is then generated by vocal synthesis. The virtual patient reacts according to the learner’s choices by displaying adaptive verbal and facial expressions of emotions.

#### Relevant Emotions and Their Expression by the Virtual Patient

We implemented the virtual patient using the MARC (Multimodal Affective and Reactive Characters) platform for animating expressive virtual agents [22]. MARC can combine nonverbal (facial and body animations) and verbal behaviors of 3D characters. The platform features a model that evaluates the current situation according to several criteria and selects facial animation accordingly [23]. This platform offers several characters that have been used in various social-skills training experiments [24,25]. We used a male and female model that suited the patient’s and his wife’s personae in the scenario.

We selected 8 emotions relevant to a suicidal crisis: sadness, anger, shame, doubt, frustration, discouragement, nervousness, and relief. We designed corresponding facial expressions using the Facial Action Coding System (Carl-Herman Hjortsjö) [26]. For sadness, anger, and shame, we used one of the multiple possibilities of action unit combinations for expressing these basic emotions [27]. For doubt, frustration, discouragement, nervousness, and relief [28], we selected the action units most frequently displayed in the 6 video clips expressing these 5 emotions.

The Acapela speech synthesizer (Acapela Group) [29] was used to generate the audio files corresponding to the spoken utterances of the virtual patient and his wife.

#### VS Scenario

We used a dialog-based scenario with a decision tree architecture in which the learner has to select the most appropriate question to ask the virtual patient at risk of suicide from a list of choices. Correct choices and distractors were selected based on usual recommendations for a psychiatric interview [20,30]. Thus, users had to pay attention to the virtual patient’s key verbal or nonverbal behaviors throughout the scenario to choose the correct question.

**Figure 1.**
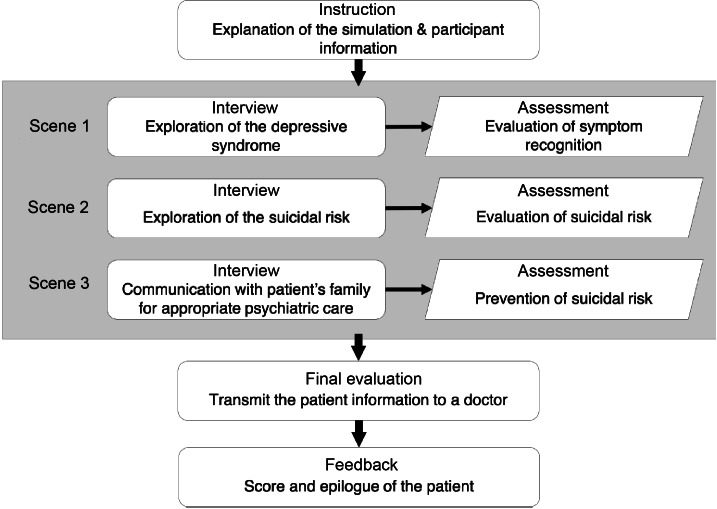
Structure of the virtual simulation scenario.

**Figure 2.**
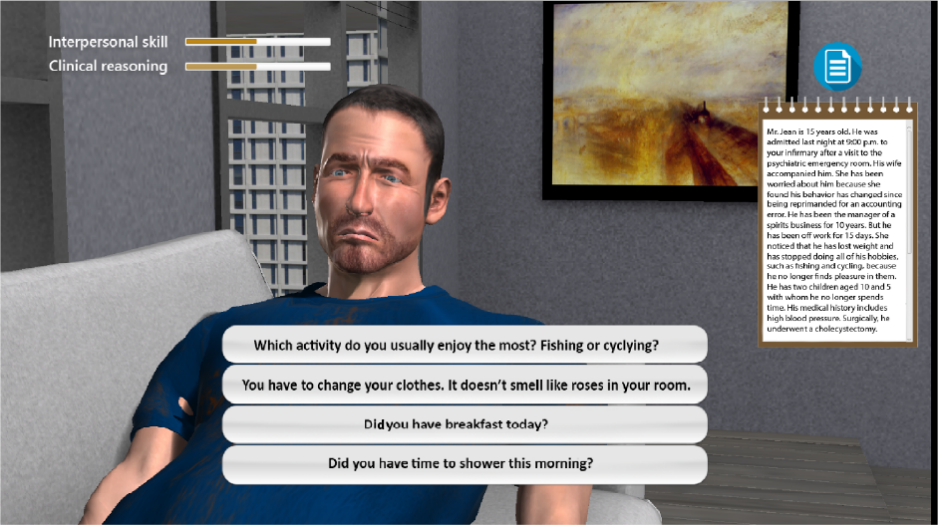
Screen capture of the interview phase.

**Figure 3.**
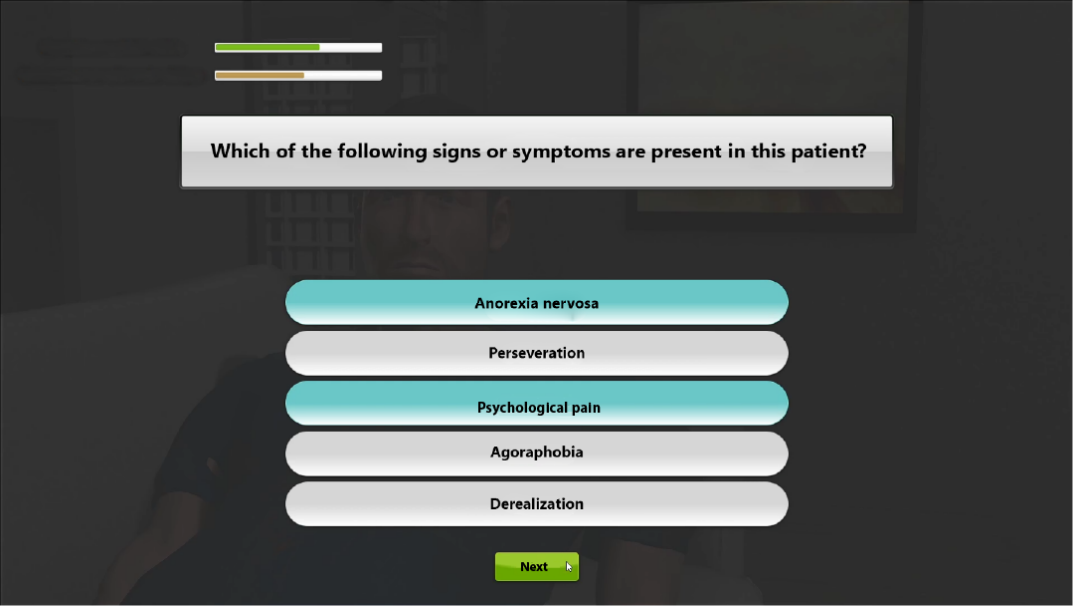
Screen capture of the assessment phase.

At the beginning of the simulation, the participants were given the instructions for the virtual interview and a nursing observation sheet describing the patient’s health status. The scenario comprised 3 scenes (see Figure 1), each with 2 phases: the interview with the virtual patient (see Figure 2) and the assessment of observed signs or symptoms (see Figure 3). The nursing observation sheet was accessible on the screen throughout the interview (see Figure 2). Multiple choice questions (MCQ) are also given during the assessment phase. After the 3 scenes, learners had to select an appropriate nursing report describing the patient’s condition based on their interviews. The feedback was given to learners at the end of the simulation based on all choices made during the simulation.

##### Design of the Interaction With the Virtual Patient

**Figure 4.**
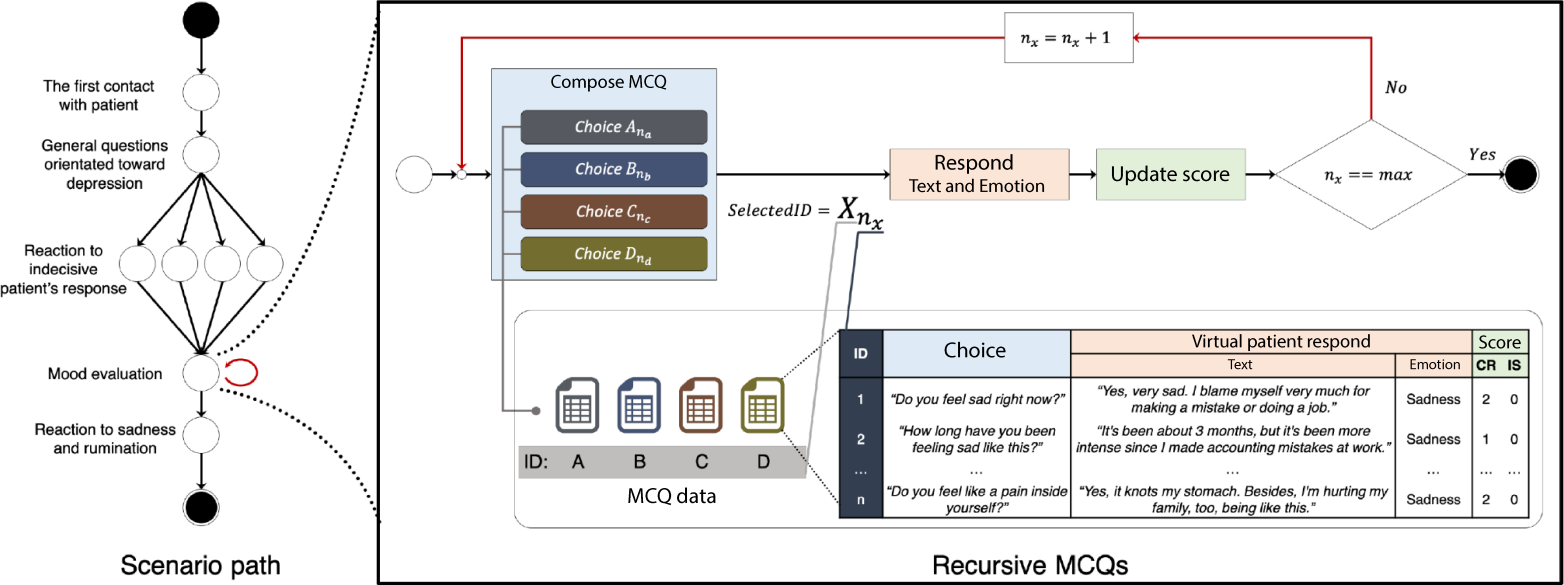
Scenario path of the first scene. Learners interview a virtual patient to diagnose the health problem: circles represent events of interviewing with multiple-choice questions format (left). Recursive multiple-choice questions occur when a set of questions needs to be asked to evaluate the patient’s health issue (right). CR: clinical reasoning; IS: interpersonal skills; MCQ: multiple choice question.

For the interview phase, we adopted a linear-interactive instructional design [31]. This design is widely used in VSs in health care and allows the learner to select available questions to diagnose and care for a patient within a formalized clinical practice model. To give learners a unique yet controlled narrative, the scenario was designed with the string of pearls model in which learners can proceed with a sequence of events based on their choice, but the main path leads to a single endpoint [32,33] (see Figure 4).

##### Multiple Choice Questions

MCQs given to participants during the screening were composed of an appropriate answer and several types of distractors:

- Psychiatric distractors investigated the semiology of obsessive-compulsive disorder, autism, and anorexia nervosa. Although some symptoms of depression might resemble these disorders (rumination and perseveration for obsessive-compulsive disorder, social withdrawal for autism, and loss of appetite for anorexia nervosa), these distractors are inadequate for the simulation of a severe, characterized depressive episode.
- Vague suicidal risk distractors could be selected by participants who wrongly thought that the use of periphrases, allusions, and hints was preferable while exploring suicidal risk to avoid distressing the patient.
- Somatic distractors were questions concerning hypertension, dysphagia, and organic asthenia, which could be plausible for the patient in this scenario. Still, they were too widely related to his actual and urgent psychiatric condition.
- Nonspecific distractors were related to family, job, and education, which are often used in a psychiatric interview but not particularly valuable for this specific situation of a psychiatric emergency.

For some events, a set of MCQs composed of an appropriate answer and 3 distractors was recursively asked of learners (Figure 4, right). Each item in the MCQ had several different questions of the same category. When the learner selected the item, only the selected item was changed in the next composed MCQ, but the other 3 items remained. The order of items was randomized each time. Once one of the items reached the last question, the user could go to the next level. Consequently, if each item had 5 different questions, the user could end the recursive MCQs after 5 to 17 loops. Throughout the simulation, 9 MCQs and 4 recursive MCQs were given during the interview phase, and 11 MCQs were provided for the assessment phase.

##### Scoring System

Learners’ performance was automatically measured according to 3 scores.

The clinical reasoning score evaluated how learners collected and interpreted signs and symptoms to determine the optimal nursing diagnosis and care plan. Clinical Reasoning was assessed during the interview and assessment phases. For example, in the interview phase, asking specific questions about suicide intention (“Have you had any suicidal thoughts in recent days?”) gave +2 points; asking vague questions about suicide intention (“Have you had any dark thoughts in recent days?”) gave +1 point; and asking a question about an irrelevant psychiatric condition (“Are you afraid of gaining weight?”) gave 0 points. The quality of the care plan and nursing clinical handover proposed by the learner had an impact on the patient’s outcome. Efficient prevention of the imminent suicidal crisis (eg “Remove shoelaces and belt, and offer uncorded pajamas”) or a good handover avoids a suicidal attempt within the psychiatric ward. By contrast, if the participant made a poor care plan (eg “Let the patient rest quietly” or “Lock him in his room”) or a poor handover, the patient attempted to commit suicide in his room, without dying, as it is usually not recommended that a patient dies within a simulation session due to the detrimental impact on the learner’s stress level and negative emotions [34].

The interpersonal skills score assessed how learners constructed a trustful and empathetic relationship with the patient. Interpersonal skills were evaluated exclusively during the interview phase. Distractors in the MCQ were selected from among untrained students’ usual mistakes in interacting with a severely depressed individual, which were collected from medical students in the third year of study during role-playing situation simulations run at the University Versailles Saint-Quentin-En-Yvelines over several years. The most common mistakes were attempts to overstimulate a slowed down and anergic patient, to cheer up a patient with pathological sadness, and nonhelpful beliefs about suicide prevention. For example, “I hear that you have suicidal thoughts and that you are desperate. We’re here to help you through this difficult time.” gave +1 points whereas “Haven’t you thought of all the harm you’ll be doing to your children who’ll be orphans?” gave −2 points.

The clinical efficiency score counted the number of questions the learner asked before the interview ended. Clinical efficiency was evaluated during the interview phase. In particular, clinical efficiency increased with the number of distractors users chose. For suicidal risk assessment, it is important to collect as much relevant information from the patient as possible to make a clinical decision in as little time as possible [35].

Clinical reasoning and interpersonal skills were scored at each MCQ and displayed with progressive score bars on the screen (see Figure 2), whereas clinical efficiency was given to the users only at the end of a sequence of recursive MCQs. During the feedback at the end of the simulation (see Figure 1), users received each score ranging from 0 (worst) to 20 (best) scaled from row scores, following the standard metric used in French education.

### Software Architecture and Procedure

**Figure 5.**
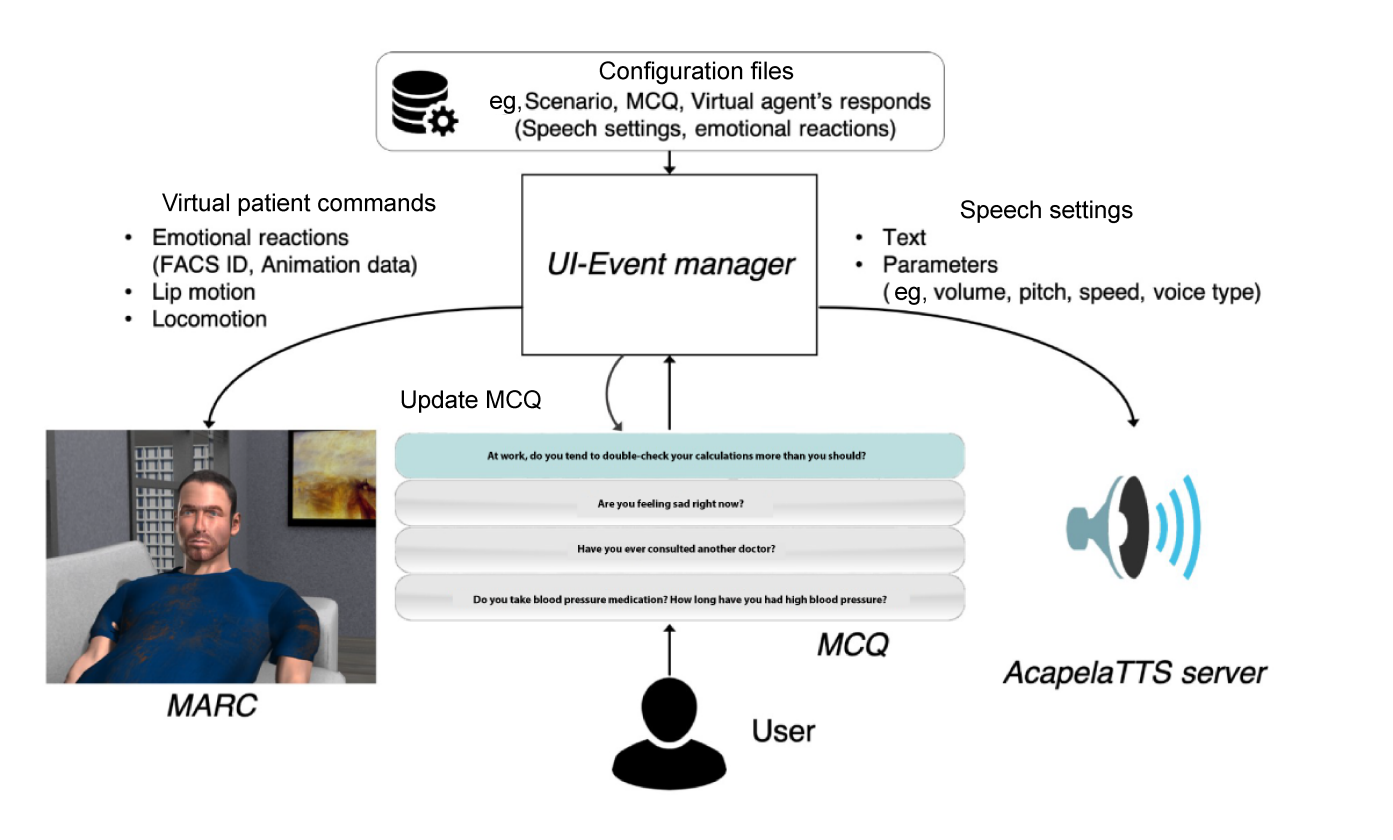
System architecture of the virtual patient simulation. MARC: Multimodal Affective and Reactive Character; MCQ: multiple choice questionnaire.

The SIVIPSY system comprises a UI-Event manager, MARC, and an AcapelaTTS server (Acapela Group; see Figure 5). The UI-Event manager is implemented in Java, updating the graphical user interface and handling each event during the simulation based on user input and a predefined scenario. The content of the scenario is defined in several external configuration files (.xml) in which the experimenter can specify the logical flow of the scenario and the MCQs that include: questions given to learners, the virtual patient’s responses, the type of emotional expression and its intensity (Action Unit combinations of each emotion are defined in the other configuration file), settings of spoken utterances (eg speed and pitch), and score settings (clinical reasoning and interpersonal skills). The UI-Event manager communicates with MARC and the AcapelaTTS server via UDP. MARC updates the virtual patient’s facial expressions and body movements based on the given requests. The AcapelaTTS server, implemented in C#, synthesizes the voice in real time with specified settings.

The learners played the simulation on a Windows PC installed at the Versailles Hospital through a remote desktop environment due to the restrictions on movement due to the COVID-19 pandemic. Remote access to the PC was handled by AnyDesk software via an internet connection, offering users remote control of the PC. The rendered resolution was 1.920×1.080 pixels, but remote users might have perceived less resolution as we prioritized the system reaction time over graphics quality in AnyDesk.

### Recruitment

Nursing students were recruited from 7 nursing schools affiliated with Versailles Saint-Quentin-En-Yvelines University. During back-to-school meetings involving all first-year students, participants were informed about the study by their nurse training manager and one author of this study (PR). Students also received an email to update them on the study. The inclusion criteria were being in the second semester of the first year of study and being over 18 years of age.

### Study Design

**Figure 6.**
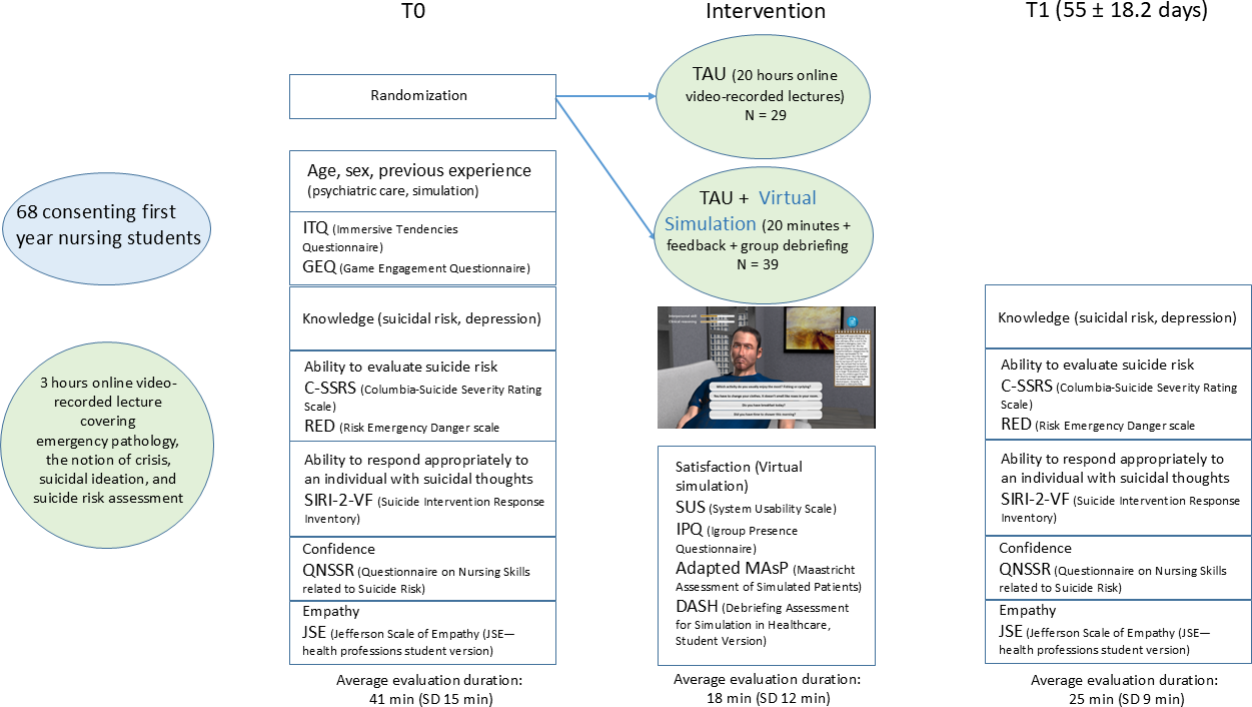
Schematic representation of the study design. TAU: teaching as usual.

The design of the study is presented in Figure 6, and the CONSORT-EHEALTH (Consolidated Standards of Reporting Trials of Electronic and Mobile Health Applications and Online Telehealth) checklist is provided in Checklist 1. No important changes were made to the methods after the investigation commenced. Participants were randomized between 2 arms with a 4:3 allocation ratio favoring VS using the “randomize” function in R (experiment package version: 1.2.1, R version 4.1.3, no random seed, simple randomization without stratification). All participants received the standard teaching program, consisting of 20 hours of online video-recorded lectures on psychiatry and mental health. Before starting the research and being randomized, participants were asked to attend a 3-hour online course covering emergency pathology, the notion of crisis, suicidal ideation, and suicide risk assessment. Randomized participants in the control arm received only the usual teaching program (teaching as usual [TAU] arm). Randomized participants in the intervention arm received TAU plus VS with SIVIPSY. Two versions of SIVIPSY were developed. In the first version, the virtual patient spoke with a neutral prosody, whereas in the second version, he spoke with a sad prosody. The 2 versions were compared with tests to the hypothesis that emotional prosody improved the perceived authenticity of the virtual patient with severe depression. All participants in 2021 were allocated to the neutral prosody version of SIVIPSY, whereas all participants in 2022 were allocated to the sad prosody version of SIVIPSY. The VS lasted approximately 20 minutes. Two types of feedback were provided to learners. The first type was automatic: the program gave 3 scores to the learners (clinical reasoning, interpersonal skills, and clinical efficiency) and revealed what happened to the patient after the interaction with the learner according to the choices that were made. The second type of feedback consisted of a remote group debriefing, co-run by a nurse and a medical teacher, when possible. It followed the rules of debriefing with good judgment [36] and was preceded by an anonymous review of the learner log file to detect performance deficits that may have varied depending on the group. The debriefing groups consisted of 4.1 individuals on average (SD 2.7), resulting in 8 different groups. Learners were grouped based on their availability.

PR generated the random allocation sequence, enrolled participants, and assigned them to interventions. Teachers and learners were not blinded. Participants were prompted by mail to engage in the evaluations, the VS, and the debriefing. The questionnaires for the 3 evaluation times were completed online by participants during several remote sessions led by PR, who addressed participant queries and ensured that all items were submitted. All questionnaires corresponding to one evaluation moment were completed at the same time for each participant. The assessment durations are reported in Figure 6.

### Sample Baseline Characteristics

Baseline characteristics were recorded at inclusion. Demographic data consisted of age, sex, and native language. We also evaluated self-reported previous experience in psychiatric care (none/[internship/employment]), previous simulation experience (no/yes), and participation in the prerequisite online course (no/yes). Finally, we evaluated the individual’s disposition to be particularly receptive to the VS modality through 2 measurements. The first consisted of their immersive tendency, measured using the Immersive Tendencies Questionnaire (ITQ) [37]. Immersion gives individuals the impression that they have left the real world and are now present in the virtual environment. A greater sense of immersion results in higher levels of involvement in a virtual environment, which may increase the pedagogical effectiveness of the VS. The second baseline disposition was the psychological engagement in video game-playing, measured using the Game Engagement Questionnaire (GEQ) [38], which may increase the positive impact of VS on pedagogical outcomes.

### Outcomes

SIVIPSY was assessed using Kirkpatrick Training Evaluation Model [39]. This model establishes the impact of a learning intervention according to five levels: (1) reaction effect: satisfaction/dissatisfaction of participants; (2) learning effect: improvement of the participants’ knowledge; (3) behavioral effect: changes in attitudes, skills, or learners’ confidence; (4) patient results (ie, whether the intervention improves the nurse diagnosis care plan) to approach operational effectiveness and reach patient-reported outcomes; and (5) return on investment. Only the first 3 Kirkpatrick levels were investigated in this study, using validated tools when available.

#### Knowledge

Knowledge was measured using a 10-item, 5-choice questionnaire on the subject of suicidal risk and depression created for the study.

#### Skills, Confidence, and Attitude

Two skills were assessed in this study, the ability to evaluate suicide risk and the ability to respond appropriately to an individual with suicidal thoughts.

##### Ability to Evaluate Suicide Risk

A specific tool was created to measure the ability to assess suicide risk in the current study. We filmed a psychiatric nurse interview of a patient with intermediate suicide risk [40]. Two academic psychiatry teachers portrayed the nurse and patient roles. The first scale used was the Columbia-Suicide Severity Rating Scale (C-SSRS), which has demonstrated good convergent and divergent validity in the international scientific literature [41].

The second scale was the Risk - Emergency - Danger (RED) scale, which is widely used in France in clinical and teaching settings [42] and recommended by the French health authorities [43]. The RED scale follows international recommendations concerning suicide risk assessment [44]. The reference for the evaluation of the suicidal crisis presented by the patient in the video was established by 11 experts, who used the 2 scales to rate the severity of the suicidal crisis (see Multimedia Appendix 1). We scored each item by computing an absolute *z* score using the recruited expert panelist’s answers as the reference and summing them for each participant for each scale.

##### Ability to Respond Appropriately to an Individual With Suicidal Thoughts

The learner’s interpersonal skills in managing suicidal crises were assessed using the Suicide Intervention Response Inventory [45], French version 2 (SIRI-2-VF) [46]. The SIRI-2-VF includes 15 statements corresponding to exchanges between patients and caregivers taken from consultation excerpts. Each exchange begins with patients’ statements concerning an aspect of their situation. Then, 2 possible answers proposed by 2 different caregivers are presented. The participant is asked to judge the suitability or unsuitability of each proposed response by giving a score ranging from −3 (very inappropriate response) to +3 (very appropriate response) on a 7-point Likert scale. We scored each excerpt by computing the absolute *z* score using published expert panelist answers [46] and summed them for each participant.

##### Confidence

We measured another behavioral effect of VS: the improvement of learners’ confidence. Confidence was measured using the Questionnaire on Nursing Skills related to Suicide Risk (QNSSR), which was adapted from a previous version initially developed for medical students [47]. It included 14 items rated on a visual analog scale ranging from the least possible confidence (coded 0) to the greatest confidence possible (coded 10, see the full scale in Multimedia Appendix 2).

##### Empathy

Empathy was measured using the Jefferson Scale of Empathy (JSE—health professions student version) [48].

### Satisfaction

Satisfaction with the serious game system user-friendliness was assessed using the System Usability Scale (SUS), one of the most widely used questionnaires to measure the perceived ease of use of interactive systems, with an excellent internal consistency (Cronbach α=0.90) [49]. Satisfaction with the immersive experience was assessed using the Igroup Presence Questionnaire (IPQ), which focuses on the subjective sense of being in a virtual environment and exhibited good internal consistency (Cronbach α=0.85 and 0.87) [50].

Satisfaction with the authenticity of the virtual patient was measured using 6 items (see Multimedia Appendix 3 extracted from the Maastricht Assessment of Simulated Patients (MAsP), which evaluates the performance of actors simulating patients in an educational setting with an acceptable internal consistency (Cronbach α=0.73) [51]. These items were chosen to be compatible with the virtual nature of the patient, as no specific scale measuring virtual patient authenticity existed at the time of testing. We also measured satisfaction with the facial expression of emotions displayed by the virtual patient using 4 items (see Multimedia Appendix 3).

Satisfaction with the remote group debriefing was assessed using the Debriefing Assessment for Simulation in Healthcare (DASH) student version [52], which showed good internal consistency with nursing students (Cronbach α=0.82) [53]. This scale explores the climate, debriefing structure, ability to engage in exchange, and strengths and areas for improvement. Finally, a 10-item questionnaire was developed to measure satisfaction with overall simulation training. It was adapted from a previous satisfaction questionnaire about simulation with medical students [47]. This questionnaire explores various aspects of satisfaction, such as the preference for simulation over another pedagogical modality, the perceived realism of the situation, and the importance of being actively involved, etc. It was completed with a question about the difficulty of the simulation and with a global measure of satisfaction on an 11-point scale.

### Statistical Analysis

Missing data were estimated using multivariate imputations by chained equations (50 imputations, mice package of R). The fraction of missing information (fmi) is reported in the results. Linear mixed-effects models were run with participants as the random factors, skills, knowledge, confidence, and empathy as the successive dependent variables, and time (2 modalities: before/after the intervention), arm (2 modalities: VS/TAU), and time-arm interaction as the simultaneous dependent variables. All analyses were run as intention-to-treat to preserve randomization. All learners randomly assigned to one of the arms were analyzed together, regardless of whether they completed the VS. The required sample size was estimated based on a meta-analysis of the educational effectiveness of simulation tools with virtual patients [54], which reports Hedges effect sizes of 0.94 for knowledge improvement. The number of subjects was estimated to be 25 in each arm, with a significance level α=.05 and power *β*=90%. A linear mixed-effects model was also run to explore the effect of the SIVIPSY version (sad or neutral vocal emotion) on satisfaction with the authenticity of the virtual patient (MaSP adapted).

### Ethical Considerations

The study was approved by the university’s ethical review board (approval number: CER-Paris-Saclay-2020‐087). Each participant provided written informed consent before inclusion. To ensure that the commitment was entirely voluntary, students were informed that their decision to participate or not would not affect the outcome of their psychiatry exam and, in turn, their progression to the second year. All study data were anonymous, but logistical measures (email confirmation and videocalls) were used to prevent participants from having multiple identities. No compensation was provided to participants for their involvement in the current research, and access to SIVIPSY was free.

## Results

### Participants

**Figure 7.**
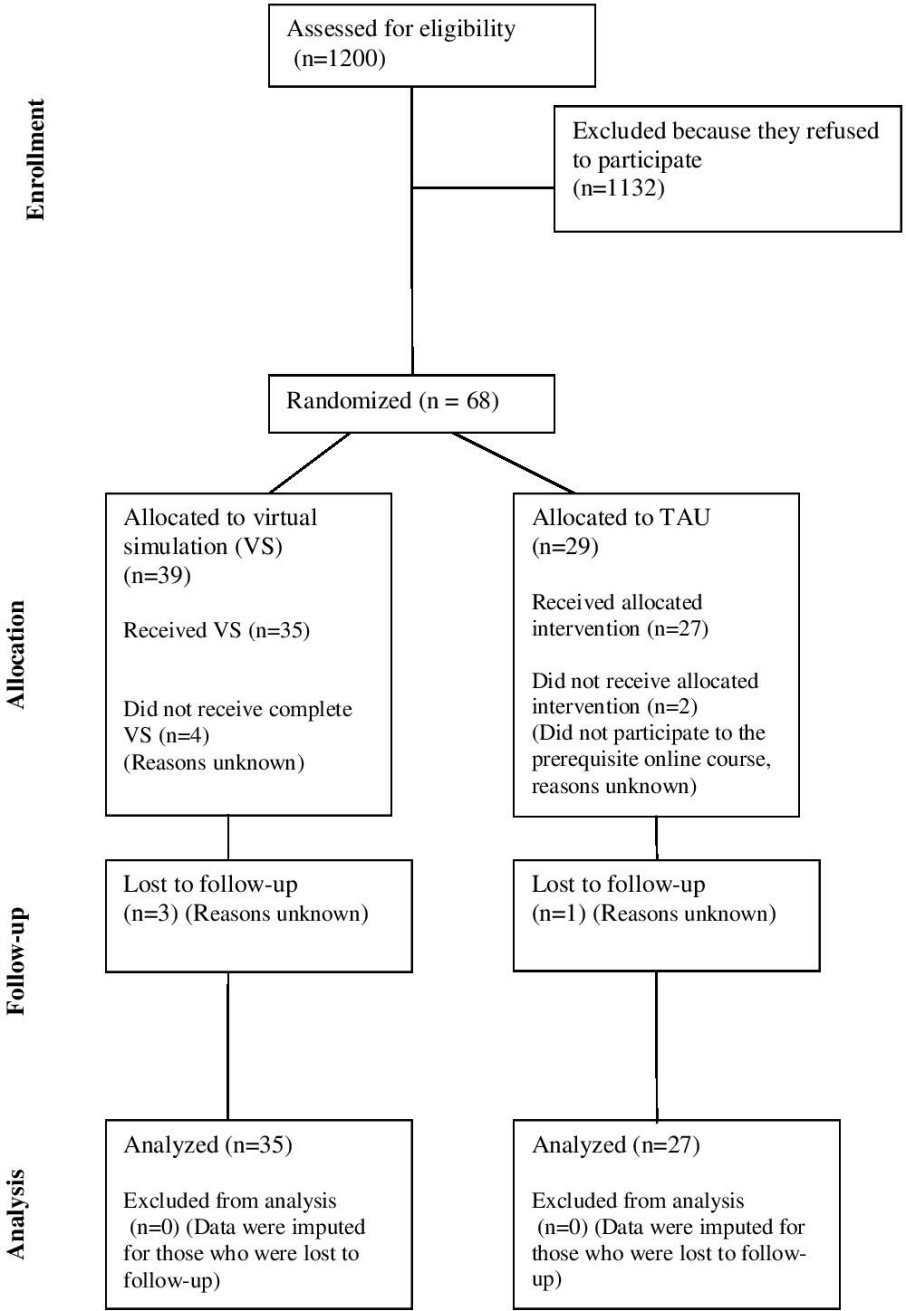
Consolidated Standards of Reporting Trial showing the flow of participants through each stage of the study. TAU: teaching as usual.

The recruitment began in February 2021 and was completed in March 2022. Approximately 1200 students were approached, but only 68 consented to participate in the study (response rate of 6%, see the CONSORT (Consolidated Standards of Reporting Trials) flow diagram of study participants in Figure 7). We included 29 students in the TAU arm and 39 in the VS arm. Most students were female, approximately 25 years of age, and few had previous experience in the psychiatric care of patients (see Table 1).

**Table 1.** Participant characteristics.

Variable	Teaching as usual (N=29)	Virtual simulation (N=39)	Statistic	*P* value
Age (years), mean (SD)	24.6 (7.9)	26.4 (10.5)	*t*_66_=−0.85	.40
Sex (female), n (%)	27 (93.1)	33 (84.6)	*χ²*_1_=0.48	.49
Native language (non-French), n (%)	2 (6.9)	4 (10.3)	*χ²*_1_=0	.96
Previous experience in psychiatric care (as an intern), n (%)	11 (37.9)	7 (17.9)	*χ²*_2_=3.57	.17
Previous experience in psychiatric care (as an employee), n (%)	3 (10.3)	4 (10.3)		
Previous simulation experience, n (%)	13 (44.8)	16 (41)	*χ²*_1_=0	.95
Participation in prerequisite online course, n (%)	27 (93)	33 (84.6)	*χ^2^*_1_=0.48	.49
Immersion (ITQ)^a^, mean (SD)	80.4 (13.3)	75.9 (17.2)	*t*_65.9_=1.2	.24
Implication (GEQ)^b^, mean (SD)	3.1 (1.4)	2.8 (1.5)	*t*_61.7_=0.78	.44

a Immersive Tendencies Questionnaire.

b Game Engagement Questionnaire.

By contrast, participants were familiar with simulation, with more than half having previous experience with this pedagogical method (either high fidelity, standardized patient, or versus). Very few participants did not study the online course before the evaluations. The average ITQ score corresponded to a moderate immersive tendency and the average GEQ score showed a slight lack of game engagement.

There were no significant statistical differences between participants in the TAU and VS arms in terms of demographic characteristics, previous psychiatric and simulation experience, participation in the prerequisite course, immersive tendency, or engagement in games (see Table 1). Four participants in the VS arm did not fully complete the intervention as intended; they were retained in the VS arm according to the intention-to-treat analysis. Among the participants in the VS arm, 18 interviewed the virtual patient with neutral prosody and 21 the virtual patient with sad prosody. No significant statistical differences were measured between participants interacting with the virtual patient expressing neutral versus sad prosody, although participants using the sad prosody version were marginally older and marginally more game-engaged (see Multimedia Appendix 4).

### Effect of the Simulation on Performance

The descriptive statistics are presented in Table 2 and the test statistics in Multimedia Appendix 4.

Outcomes were measured at an average of 55 (SD 18.2) days after inclusion. There were no missing data for TAU before the intervention, 3% of missing data for TAU after the intervention, no missing data for VS before the intervention, and 8% of missing data for VS after the intervention.

**Table 2.** Performance (skills, knowledge, confidence, and empathy) in the 2 arms before and after the simulation.

Variable		Teaching as usual		Virtual simulation		Effect size
	Theoretical range	Before, mean (SD)	After, mean (SD)	Before, mean (SD)	After, mean (SD)	Cohen *d* (95% CI)
C-SSR^a^ (sum of absolute *z scores)*	1.1‐16.7	3.6 (1.6)	3.7 (2.4)	3.6 (1.7)	3 (1.6)	−0.2 (−0.7 to 0.29)
RED^b^ (sum of absolute *z* scores)	1.1‐6.3	3 (1.4)	3.5 (1.8)	2.9 (1.5)	3.2 (1.5)	−0.17 (−0.67 to 0.32)
SIRI^c^ (sum of absolute *z* scores)	10.8‐188	44.8 (15.8)	43.2 (15.9)	46.7 (14.1)	40.3 (11.9)	−0.44 (−0.94 to 0.06)
Knowledge (total)	0‐50	35.7 (4.8)	35.7 (4.4)	37.8 (3.9)	38.1 (4)	0.03 (−0.46 to 0.53)
QNSSR^d^ (total)	0‐100	56.7 (13.2)	59.7 (15.1)	51 (10.8)	61.6 (12.4)	0.81 (0.29-1.32)
JSE^e^ (total)	20‐140	108.1 (9.5)	107 (9.8)	110.3 (8.7)	110.7 (7.2)	0.06 (−0.43 to 0.56)

a C-SSR: Columbia Suicide Severity Rating Scale.

b RED: Risk-Emergency-Danger scale.

c SIRI: Suicide Intervention Response Inventory.

d QNSSR: Questionnaire on Nursing Skills related to Suicide Risk.

e JSE: Jefferson Scale of Empathy (Health Professions Student version).

#### Knowledge

The effect of the time:arm interaction on knowledge was nonsignificant (0 points of increase after TAU versus 0.3 points of increase after VS, B=0.2; SE 0.8; *t*_119.7_=0.2; *P*=.82; fmi=0.084), suggesting that the intervention was ineffective in improving knowledge of suicidology.

#### Skills, Attitude, and Confidence

##### Ability to Evaluate Suicide Risk

The effect of the time, arm interaction on the C-SSRS (0.1 points of worsening after TAU versus 0.3 points of improvement after VS, B=−0.3; SE 0.5; *t*_125.6_=−0.6; *P*=.58; fmi=0.047) and RED scales (0.5 points of worsening after TAU vs 0.3 points of worsening after VS, B=−0.3; SE 0.5; *t*_124.8_=−0.5; *P*=.59; fmi=0.052) was nonsignificant, suggesting that the intervention was ineffective in improving the evaluation of suicide risk.

##### Ability to Respond Appropriately to an Individual With Suicidal Thoughts

The effect of the time, arm interaction on the SIRI was marginally significant (1.6 points of improvement after TAU versus 6.4 points of improvement after VS, B=−4.5; SE 2.5; *t*_119.5_=−1.8; *P*=.08; fmi=0.085). The time effect was nonsignificant for the TAU (B=−1.6; SE 1.8; *t*_50.9_=−0.9; *P*=.36; fmi=0.09), whereas it was significant for the VS (B=−6.2; SE 1.8; *t*_64.9_=−3.5; *P*=.001; fmi=0.136). The performance in response to suicidal thoughts improved after VS, suggesting a possible effectiveness of the intervention on how to interact with a patient experiencing a suicidal crisis.

##### Confidence

The effect of the time:arm interaction on the QNSSR was significant (3 points of increase after TAU versus 10.6 points of increase after VS, B=7.2; SE 2.5; *t*_111.5_=2.8; *P*=.006; fmi=0.131). The time effect was marginally significant for the TAU (B=3; SE 1.8; *t*_50.9_=1.7; *P*=.097; fmi=0.091) and significant for the VS (B=10.2; SE 1.7; *t*_62.8_=5.8; *P*<.001; fmi=0.159). This suggests the effectiveness of the versus in increasing confidence in one’s ability to interact with a patient experiencing a suicidal crisis.

### Empathy

The effect of the time:arm interaction on knowledge was nonsignificant (1.1 points of worsening after TAU vs 0.4 points of improvement after VS, B=1.2; SE 2; *t*_122.9_=0.6; *P*=.54; fmi=0.065), suggesting that the intervention was ineffective in improving empathy.

### Satisfaction

The satisfaction results are presented in Table 3.

Internal consistency was excellent for the DASH, good for IPQ and the virtual patient facial emotions quality, acceptable for the virtual patient authenticity, questionable for the satisfaction with overall simulation training, and poor for the SUS. The user-friendliness of SIVIPSY was very high. The simulation induced a moderate sense of presence. The virtual patient was considered relatively authentic, with appropriate natural facial expressions of emotions, neither inhibited nor exaggerated. The debriefing was considered to be very good to excellent, and satisfaction with the overall intervention was between good and very good. The difficulty of the scenario was considered appropriate.

**Table 3.** Satisfaction with the virtual simulation.

Variable	Theoretical range	Mean (SD)	Missing (%)	Cronbach α
User-friendliness				
System Usability Scale	0-100	87.1 (9.9)	10.3	0.54
Overall friendliness	1‐7	5.5 (0.7)	10.3	—
Presence				
Igroup Presence Questionnaire (item average)	1-7	4.2 (1)	10.3	0.84
Satisfaction with the virtual patient				
Authenticity (item average)	1-4	3.1 (0.6)	10.3	0.79
Facial emotions quality (item average)	1‐4	3 (0.7)	12.8	0.89
Facial emotions quantity	1‐5	3 (0.9)	12.8	—
Debriefing				
DASH^b^ total (item average)	1-7	6.5 (0.5)	12.8	0.96
DASH stage setting (item average)	1‐7	6.5 (0.5)	12.8	0.83
DASH learning context (item average)	1‐7	6.6 (0.4)	12.8	0.79
DASH organized debriefing (item average)	1‐7	6.6 (0.5)	12.8	0.83
DASH reflection on performance (item average)	1‐7	6.3 (0.6)	12.8	0.81
DASH performance identification (item average)	1‐7	6.3 (0.8)	12.8	0.91
DASH help to improve (item average)	1‐7	6.4 (0.7)	12.8	0.81
Satisfaction with the overall simulation training				
Questionnaire (item average)	1-4	3.4 (0.3)	12.8	0.62
Perception of difficulty	1‐5	2.9 (0.3)	12.8	—
Global rating	0‐10	8.7 (1)	12.8	—

a Not applicable.

b DASH: Debriefing Assessment for Simulation in Healthcare.

### Impact of Emotional Prosody on Satisfaction With the Virtual Patient

**Figure 8.**
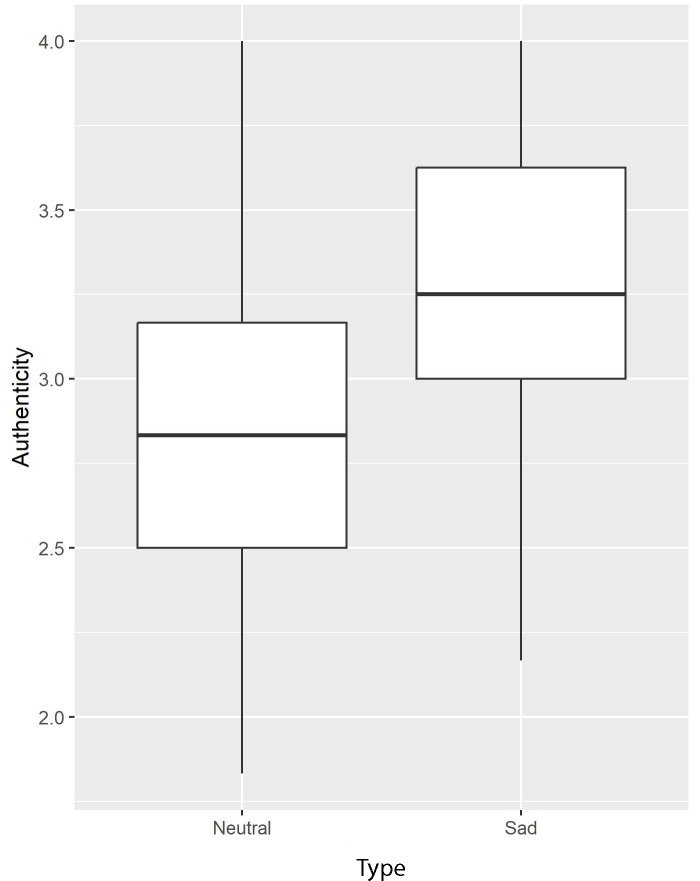
Plot of the virtual patient’s authenticity according to his prosody.

The effect of Prosody on virtual patient authenticity (MASP adapted) was significant (B=0.4; SE 0.2; *t*_71_=2.2; *P*=.03; fmi=0.065). Learners rated the virtual patient as more authentic when he had a sad prosody than a neutral prosody (see Figure 8). This difference became marginally significant when age and GEQ (which were marginally different between the 2 conditions) were entered as covariates in the model (B=0.4; SE 0.2; *t*_63.7_=1.9; *P*=.06; fmi=0.131).

## Discussion

### Overview

This exploratory study aimed to evaluate the effectiveness of VS in training nursing students in suicide risk assessment through a randomized controlled investigation. Pedagogical effectiveness was measured in terms of satisfaction, knowledge, skills, confidence, and empathy. The VS group exhibited higher confidence and a better ability to respond appropriately to suicidal thoughts after VS compared with the control group. Satisfaction with VS was high, particularly regarding the authenticity of the virtual patient. This authenticity was perceived as greater when emotional prosody was included alongside facial emotions.

### Principal Findings

SIVIPSY was more efficient than usual teaching to improve confidence in nursing skills for the assessment and care of an individual experiencing a suicidal crisis. The picture was less clear in terms of the effectiveness of SIVIPSY in improving nursing skills related to suicidal crises. The ability to respond appropriately to an individual with suicidal thoughts significantly increased after the VS, whereas it did not after the usual teaching. However, the difference in improvement after VS and TAU was only marginally significant, suggesting that further studies are needed to confirm the greater effectiveness of VS in improving adequate nursing responses to individuals experiencing a suicidal crisis.

By contrast, SIVIPSY was not associated with an improvement in the ability to assess the severity of a suicidal crisis. SIVIPSY may thus be ineffective in improving the accuracy of suicide risk assessment. Another explanation could be a lack of sensitivity to change in the tool we have developed to measure this construct. SIVIPSY was also not associated with knowledge improvement. Neither VS nor TAU modified nursing students’ general level of empathy. The average empathy score in the present sample was comparable to that reported in a US sample [48]. Empathy measured with the JSE may be a relatively stable personality disposition, even in the health care setting [55], which is unlikely to change after a one-off intervention such as a simulation session. Changes in empathy have been observed in medical students, but only over several years, with a progressive decline, especially after having spent more time interacting with patients [56]. The existence of such a decline needs to be clarified concerning nursing students, for which there are conflicting results [57,58].

There was high satisfaction with SIVIPSY user-friendliness and the overall simulation, although these results may lack reliability due to poor and questionable internal consistencies found for these questionnaires. The satisfaction with the group debriefing was very high and very reliable, suggesting that SIVIPSY should be implemented in teaching as usual. A social desirability bias could explain the high level of satisfaction reported in this study, as academic teachers were involved in the debriefing. However, all questionnaires were anonymously completed, which should have limited such bias. The level of satisfaction was reliable and good concerning the sense of presence, the authenticity of the virtual patient, and his emotional reactions. Our results suggest that authenticity was, as expected, improved using emotional prosody, although such improvement could be partially explained by the older age and greater game implication of the participants who used the sad prosody version of the VS. Future virtual patients in pedagogical settings should display vocal expression of emotions, as authenticity is a critical component of interactive multimedia mental health education programs for nurses [59].

### Comparison to Previous Work

A previous study reported that nursing students had higher self-confidence after a simulated standardized patient session than those who watched a traditional video-recorded lecture on suicide [27]. Our results suggest that the beneficial impact of simulation involving actors playing simulated patients extends to VS. It is reasonable to assume that changes in attitudes with the improvement of confidence favored by VS may improve suicidal crisis detection, given that attitudes and self-efficacy are associated with the intention to engage in suicide prevention behaviors [60]. Nursing students often feel anxiety and fear before they start their clinical practice in psychiatry [61]. One reason for them to avoid evaluating suicide risk is the anticipation of not knowing what to do in case of suicidal thoughts. Enhancing confidence may have a positive impact on practical behavior (with an increase in suicide risk assessment of actual patients) and lead to improvements in attitude and reduce stigma [3].

Regarding the lack of knowledge improvement after VS, a previous study also reported that knowledge about suicide was comparable after simulation with a standardized patient and after a traditional lecture on suicide [29]. This suggests that the specificity of the pedagogical action of simulation does not appear to lie in improving theoretical nursing knowledge. A recent analysis confirmed this interpretation by reporting a lack of significant differences between simulation, in general, and controls in improving suicide risk assessment and intervention in RCTs, while non-RCT interventions with simulation and pre-post design were associated with knowledge improvement [62].

### Strengths and Limitations

The 2 main strengths of this study are the innovative use of an emotionally reactive virtual patient to train students in caring for patients with suicidal ideation and the randomized controlled design of the intervention. The main methodological limitation of this study was the lack of an active comparator (an additional pedagogical intervention in the control arm), which may have inflated the efficiency of VS. As previously reported, attitudes (ways of approaching patients with suicidal ideas, beliefs, self-efficacy, confidence, and sense of preparedness regarding suicidal crises) improved significantly when simulation training was compared to an inactive comparator. By contrast, no significant difference was found concerning these attitudes when simulation training was compared to an active comparator [62]. Other methodological limitations were the lack of preregistration for this exploratory study, with no primary outcome selected, and the lack of a control for multiple comparisons. Despite randomization, groups were unbalanced in terms of the outcomes, with less confidence and a lower ability to respond appropriately to an individual with suicidal thoughts in the intervention group than the control group. Further studies should confirm the present results by stratifying learners based on these 2 outcomes. Finally, the expressiveness of the virtual patient is still limited, particularly postures and gestures.

### Future Directions

Further simulation may benefit from increasing online feedback during the interaction with the virtual patient beyond the evolution of bar scores implemented in SIVIPSY. It has been reported that per-interview feedback increased clinicians’ empathetic responses toward a patient experiencing a suicide crisis more than postinterview feedback during training with a virtual patient [63]. The time dedicated to coding the verbal and emotional reactions of the virtual patient was substantial, and further interventions should use a more dynamic approach to emotional expressions based, for example, on the appraisal theory of emotion [64,65] to automate the avatar reactions. This could increase the authenticity of the virtual patient and decrease the burden on the teacher who creates the simulation scenario. Future studies should include an active comparator in the control arm, which may consist of a teaching program without simulation (such as recorded lectures on suicide assessment, delivery of booklets followed by question-and-answer discussions, or role plays) or with simulation using an alternative method (like simulation with standardized patients or expert patients).

Our findings have significant implications for disseminating and implementing SIVIPSY in nursing curricula. The intervention, which improved the management of individuals exhibiting suicidal behaviors, may help reduce unnecessary emergency room visits in the context of a marked deterioration in the mental health of young women in France after the COVID-19 pandemic [66]. The pedagogical effectiveness of VS reported in this study may translate into clinical efficiency, improving patient-reported outcomes, such as reducing suicide attempts and mortality by suicide following the implementation of this simulation training program. One argument favoring this transfer arises from the fact that training primary care doctors and nurses to better screen and treat depression, with supplemental help from psychiatrists, lowered suicide rates, suicide attempts, and suicidal ideation [67]. However, the studies investigating the clinical efficiency of suicide prevention programs often combine education, training, screening, and referral for treatment. The separate effect of each intervention component remains unknown, and further studies should assess the specific clinical efficiency of suicide risk training programs. For instance, long-term patient outcomes should be investigated after the nurses enter clinical practice, such as the number of structured suicide risk screenings conducted in clinical practice, which should increase after the VS training, or the number of suicide attempts, which should decrease. An operational efficiency of the VS would also imply a return on investment by reducing suicidality-related costs.

### Conclusions

In conclusion, VS shows encouraging results in improving the confidence and skills of nursing students to behave appropriately with patients in suicidal crises. Further studies are needed to assess the impact of VS on patient outcomes such as the prevention of death by suicide and suicide attempts and the return on training investment.

## Supplementary material

10.2196/69347 Multimedia Appendix 1 Establishment of the scoring norms for the Columbia-Suicide Severity Rating Scale and the Risk-Emergency-Danger tool.

10.2196/69347 Multimedia Appendix 2 Questionnaire on Nursing Skills related to Suicide Risk (QNSSR).

10.2196/69347 Multimedia Appendix 3 Satisfaction questionnaires.

10.2196/69347 Multimedia Appendix 4 Test statistics of the effect of the simulation on performance.

10.2196/69347 Multimedia Appendix 5 Additional material.

10.2196/69347 Checklist 1 CONSORT-eHEALTH checklist (V 1.6.1).

## Abbreviations

C-SSRS: Columbia-Suicide Severity Rating Scale
CONSORT: Consolidated Standards of Reporting Trials
CONSORT-EHEALTH: Consolidated Standards of Reporting Trials of Electronic and Mobile Health Applications and Online Telehealth
DASH: Debriefing Assessment for Simulation in Healthcare
fmi: fraction of missing information
GEQ: Game Engagement Questionnaire
IPQ: Igroup Presence Questionnaire
ITQ: Immersive Tendencies Questionnaire
JSE: Jefferson Scale of Empathy
MARC: Multimodal Affective and Reactive Characters
MAsP: Maastricht Assessment of Simulated Patients
MCQ: multiple choice question
QNSSR: Questionnaire on Nursing Skills related to Suicide Risk
RED: Risk - Emergency - Danger
SIRI-2-VF: Suicide Intervention Response Inventory, French version 2
SIVIPSY: Simulation with a Virtual Patient in Psychiatry
SUS: System Usability Scale
TAU: teaching as usual
VS: virtual simulation
