# Supplementary material for: Effectiveness of a Web-Based Virtual Simulation to Train Nursing Students in Suicide Risk Assessment: Randomized Controlled Investigation
Source: JMIR Serious Games. 2025 Aug 1;13:e69347. doi: 10.2196/69347 (PMC12316442; doi:10.2196/69347)
Supplement: Multimedia Appendix 1 [file games-v13-e69347-s001.docx]

**Multimedia Appendix 2.** Establishment of the scoring norms for the Columbia-Suicide Severity Rating Scale and the Risk-Emergency-Danger tool.

To establish references for the evaluation of the suicidal crisis of the patient presented in the video, 11 experts watched the filmed interview and rated the different items of the Columbia-Suicide Severity Rating Scale (CSSR-S) and the Risk-Emergency-Danger scale (RED). Their mean age was 38.3 (sd: 7.6) years. There were 10 psychiatrists and one nurse. There were two males. The mean duration of professional experience was 9.4 (sd: 7.6) years.

The CSSR-S includes three items. In the first item, the participant is asked to judge the severity of suicidal ideation by choosing one of the following levels:

- no wish to be dead, giving a score of 0
- wish to be dead (subject endorses thoughts about a wish to be dead or not alive anymore, or wish to fall asleep and not wake up), giving a score of 1
- non-specific active suicidal thoughts (general non-specific thoughts of wanting to end one’s life/commit suicide), giving a score of 2
- active suicidal ideation with any methods (not plan) without intent to act (subject endorses thoughts of suicide and has thought of at least one method during the assessment period. This is different than a specific plan with time, place, or method details worked out), giving a score of 3
- active suicidal ideation with some intent to act, without a specific plan (active suicidal thoughts of killing oneself and the subject reports having some intent to act on such thoughts), giving a score of 4
- active suicidal Ideation with specific plan and intent (thoughts of killing oneself with details of plan fully or partially worked out and the subject has some intent to carry it out), giving a score of 5.

The mean score for the severity of “Suicidal Ideation” was 3.9 (sd: 0.8), corresponding to suicidal intent without a specific plan.

In the second item, the participant is asked to evaluate the intensity of suicidal ideation by assessing deterrents according to the following levels:

- deterrents definitely stopped the patient from attempting suicide, giving a score of 1
- deterrents probably stopped the patient, giving a score of 2
- uncertain that deterrents stopped the patient, giving a score of 3
- deterrents most likely did not stop the patient, giving a score of 4
- deterrents definitely did not stop the patient, giving a score of 5

The mean score for “Intensity of Ideation - Deterrents” was 1.6 (sd: 0.5), corresponding to a situation between "Deterrents definitely stopped the patient from attempting suicide" and "Deterrents probably stopped the patient".

In the third item, the participant is asked to evaluate the intensity of suicidal ideation by assessing the reasons for ideation according to the following levels:

- completely to get attention, revenge, or a reaction from others, giving a score of 1
- mostly to get attention, revenge, or a reaction from others, giving a score of 2
- equally to get attention, revenge, or a reaction from others and to end/stop the pain, giving a score of 3
- mostly to end or stop the pain (the patient couldn’t go on living with the pain or how he was feeling), giving a score of 4
- completely to end or stop the pain (the patient couldn’t go on living with the pain or how he was feeling), giving a score of 5

The mean score for “Intensity of Ideation - Reasons for Ideation” was 4.1 (sd: 0.3), corresponding to "Mostly to end or stop the pain". The three other dimensions of the “Intensity of Ideation” (frequency, duration, and controllability) were not investigated during the filmed interview and were thus discarded.

Regarding the RED scale, the first item evaluates suicide risk factors according to the following levels: low (coded as 1), intermediate (coded as 2), or high (coded as 3). The second item evaluates the suicidal emergency (weak, coded as 1; intermediate, coded as 2; high, coded as 3; and imminent, coded as 4), and the third item the dangerousness of the suicidal ideation (low, coded as 1; intermediate, coded as 2; high, coded as 3). The mean suicidal risk level was 2.4 (sd: 0.5), corresponding to a moderate to elevated risk. The mean suicidal emergency level was 2.3 (sd: 0.6), corresponding to a moderate to elevated emergency. The mean suicidal danger level was 2 (sd: 0.6), corresponding to a moderate danger.
