# Supplementary material for: Effectiveness of a Web-Based Virtual Simulation to Train Nursing Students in Suicide Risk Assessment: Randomized Controlled Investigation
Source: JMIR Serious Games. 2025 Aug 1;13:e69347. doi: 10.2196/69347 (PMC12316442; doi:10.2196/69347)

**Multimedia Appendix 3.** Questionnaire on Nursing Skills related to Suicide Risk (QNSSR).

The following questionnaire measures your confidence in your ability to assess suicidal risk. It comprises 14 questions. To give your answers, you'll use a scale with a cursor that you can move between 0 and 10.

1. On a scale of 0 to 10, what is the level of your theoretical knowledge in the field of suicidal risk and behavior?

Please drag the cursor to give your answer.

0 for least knowledge, 10 for most knowledge


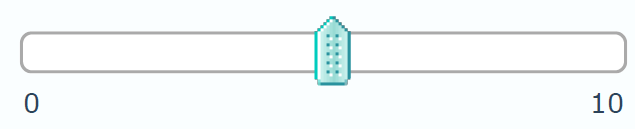


1. On a scale of 0 to 10, how competent do you feel in clinical reasoning about suicidal risk and behavior?

Please drag the cursor to give your answer.

0 for least skill, 10 for most skill


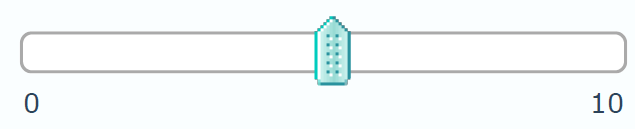


1. On a scale of 0 to 10, how competent do you feel in assessing a person presenting suicidal ideation?

Please drag the cursor to give your answer.

0 for least skill, 10 for most skill


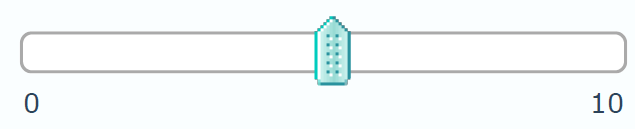


1. On a scale of 0 to 10, how anxious do you feel about assessing someone with suicidal ideation?

Please drag the cursor to give your answer.

0 for least anxiety, 10 for most anxiety


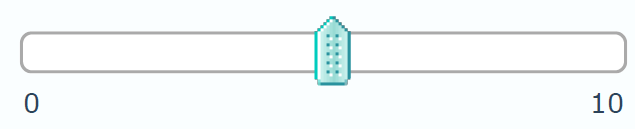


1. On a scale of 0 to 10, how confident are you in your ability to set priorities during a suicide risk assessment interview?

Please drag the cursor to give your answer.

0 for least confidence, 10 for most confidence


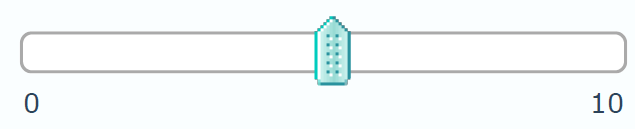


1. On a scale of 0 to 10, how confident are you in your communication skills during a suicide risk assessment interview?

Please drag the cursor to give your answer.

0 for least confidence, 10 for most confidence


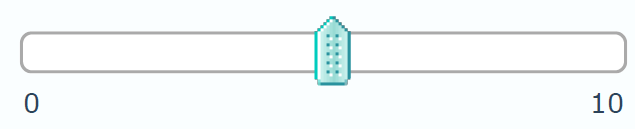


1. On a scale of 0 to 10, how competent do you feel in assessing someone who has just attempted suicide?

Please drag the cursor to give your answer.

0 for least skill, 10 for most skill


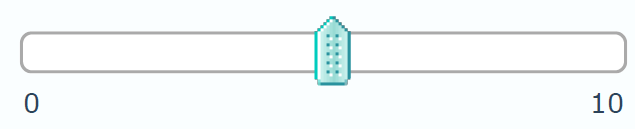


1. On a scale of 0 to 10, how anxious do you feel about assessing someone who has just attempted suicide?

Please drag the cursor to give your answer.

0 for least anxiety, 10 for most anxiety


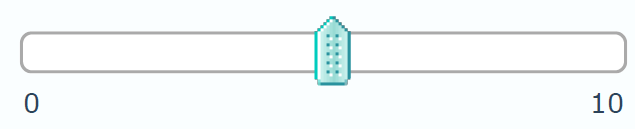


1. On a scale of 0 to 10, how confident do you feel in anticipating problems during the care of a patient presenting a suicidal crisis?

Please drag the cursor to give your answer.

0 for least confidence, 10 for most confidence


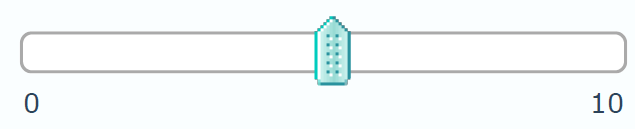


1. On a scale of 0 to 10, how confident do you feel in your ability to exchange psychiatric information with a patient experiencing a suicidal crisis, their family and friends, and your healthcare colleagues?

Please drag the cursor to give your answer.

0 for least confidence, 10 for most confidence


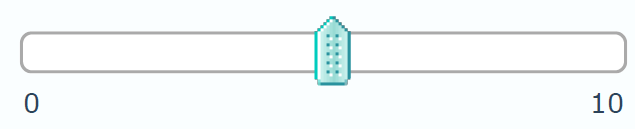


1. On a scale of 0 to 10, how competent do you feel in caring for a person presenting suicidal ideation?

Please drag the cursor to give your answer.

0 for least competent, 10 for most competent


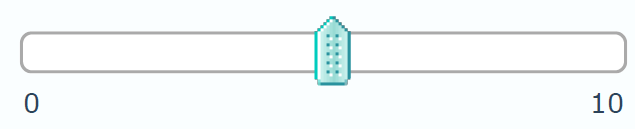


1. On a scale of 0 to 10, how anxious do you feel about caring for someone with suicidal thoughts?

Please drag the cursor to give your answer.

0 for least anxiety, 10 for most anxiety


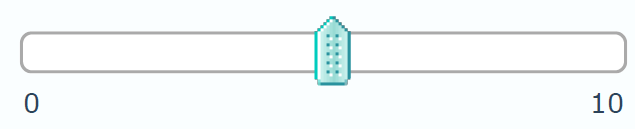


1. On a scale of 0 to 10, how competent do you feel in dealing with someone who has just attempted suicide?

Please drag the cursor to give your answer.

0 for least competent, 10 for most competent


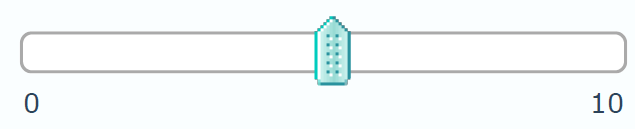


1. On a scale of 0 to 10, how anxious do you feel about caring for someone who has just attempted suicide?

Please drag the cursor to give your answer.

0 for least anxiety, 10 for most anxiety


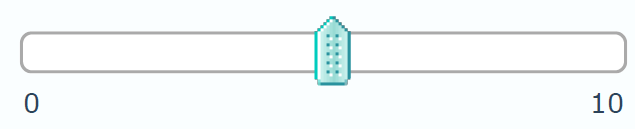

Supplement: Multimedia Appendix 2 [file games-v13-e69347-s002.docx]
