# Supplementary material for: Effectiveness of a Web-Based Virtual Simulation to Train Nursing Students in Suicide Risk Assessment: Randomized Controlled Investigation
Source: JMIR Serious Games. 2025 Aug 1;13:e69347. doi: 10.2196/69347 (PMC12316442; doi:10.2196/69347)
Supplement: Multimedia Appendix 3 [file games-v13-e69347-s003.docx]

**Multimedia Appendix 4.** Satisfaction questionnaires.

**Authenticity of the virtual patient** (items selected from the Maastricht Assessment of Simulated Patients (Wind et al., 2004) to assess the virtual patient authenticity)

|  | Complete disagreement | Moderate disagreement | Moderate agreement | Complete agreement |
| --- | --- | --- | --- | --- |
| SP appears authentic |  |  |  |  |
| SP might be a real patient |  |  |  |  |
| SP is challenging/testing the student |  |  |  |  |
| SP simulates physical complaints unrealistically |  |  |  |  |
| SP's appearance fits the role |  |  |  |  |
| SP answers questions in a natural manner |  |  |  |  |

**Quality of the facial emotions expressed by the virtual patient**

| The emotional expressions expressed by the virtual patient's face are | Very inconsistent | Moderately inconsistent | Moderately consistent | Very consistent |
| --- | --- | --- | --- | --- |
|  | Very fake | Moderately fake | Moderately realistic | Very realistic |
|  | Very artificial | Moderately artificial | Moderately natural | Very natural |

**Quantity of the facial emotions expressed by the virtual patient**

| The emotional expressions expressed by the virtual patient's face are | Very inhibited | Moderately inhibited | Neither inhibited nor exagerated | Moderately exagerated | Very  exagerated |
| --- | --- | --- | --- | --- | --- |

**Satisfaction with the overall simulation training**

| How much do you think this simulation-based psychiatry training will improve your future practice? | Very little | Little | Much | Very much |
| --- | --- | --- | --- | --- |
| Would you have preferred to complete your training in psychiatry with a lecture instead of simulation? | Completely  agree | Agree | Disagree | Completely  disagree |
| Would you have preferred role-playing (with some students playing the role of patients) to simulation teaching to complete your psychiatry training? | Completely  agree | Agree | Disagree | Completely  disagree |
| How do you see this simulation session in relation to the training you received during your psychiatric internship? | Much worse | Worse | Better | Much better |
| How do you situate this simulation session in relation to the psychiatry teaching you received during the mangistral online courses? | Much worse | Worse | Better | Much better |
| How important do you think it is to continue simulation teaching next year? | of very little importance | of little importance | important | very important |
| How important do you think it is to make simulation teaching compulsory next year? | of very little importance | of little importance | important | very important |
| How much did participating in the simulation help you learn? | Very little | Little | Much | Very much |
| How realistic did you find the setting? | Very little | Little | Much | Very much |
| How much did the debriefing session help you in your learning process? | Very little | Little | Much | Very much |

| **Evaluate the level of difficulty of the simulation scenario** | Much too easy | Too easy | Appropriate | Too difficult | Much too difficult |
| --- | --- | --- | --- | --- | --- |
