# Supplementary material for: Effectiveness of a Web-Based Virtual Simulation to Train Nursing Students in Suicide Risk Assessment: Randomized Controlled Investigation
Source: JMIR Serious Games. 2025 Aug 1;13:e69347. doi: 10.2196/69347 (PMC12316442; doi:10.2196/69347)
Supplement: Multimedia Appendix 4 [file games-v13-e69347-s004.docx]

**Multimedia Appendix 4.** Participant characteristics according to the virtual patient's prosody.

|  | Neutral prosody  (N = 18) | Sad prosody  (N = 21) |  |  |
| --- | --- | --- | --- | --- |
|  | Mean (sd) or % | Mean (sd) or % | Statistic | p |
| Age (years) | 23.1 (8.4) | 29.3 (11.4) | t(36.3) = -1.97 | 0.056 |
| Sex | 83.3 (Female) | 85.7 (Female) | Chi(1) = 0 | 1 |
| Native language | 16.7 (Non French) | 4.8 (Non French) | Chi(1) = 0.48 | 0.489 |
| Previous experience in psychiatric care (as an intern, as an employee, or with no experience) | 16.7 (intern) | 19 (intern) | Chi(2) = 0.92 | 0.632 |
|  | 5.6 (employed) | 14.3 (employed) |  |  |
| Previous simulation experience | 55.6 (No) | 61.9 (No) | Chi(1) = 0.01 | 0.94 |
| Participation in prerequisite online course | 22.2 (N) | 9.5 (N) | Chi(1) = 0.42 | 0.515 |
| Immersion (ITQ) | 79.2 (13.2) | 73.2 (19.9) | t(35) = 1.12 | 0.271 |
| Implication (GEQ) | 2.4 (1.1) | 3.3 (1.6) | t(35.8) = -2 | 0.054 |
